# Silicone oil-associated visual loss in macula-on rhegmatogenous retinal detachment – does the tamponade duration affect the outcome?

**DOI:** 10.1186/s12886-026-05235-4

**Published:** 2026-08-17

**Authors:** Teresa Barth, Horst Helbig, Maria-Andreea Gamulescu, David Märker

**Affiliations:** Department of Ophthalmology, University Medical Centre Regensburg, Franz-Josef-Strauß-Allee 11, Regensburg, 93053 Germany

**Keywords:** Silicone oil, Macula-on retinal detachment, Unexplained visual loss - endotamponade duration

## Abstract

**Background:**

Silicone oil (SO)-associated unexplained visual loss (UVL) is a serious phenomenon after pars-plana-vitrectomy (ppV). Due to reports of UVL, we started earlier SO removal (SOR) in macula-on rhegmatogenous retinal detachments (RRD) in 2020. This study aims to investigate the effect of a shorter (≤ 60 days) compared to a long-term (> 60 days) SO tamponade.

**Methods:**

All consecutive macula-on RRDs (2020–2025) treated with ppV were retrospectively analysed comparing eyes with short-term vs. long-term SO tamponade regarding best corrected visual acuity (BCVA), intraocular pressure (IOP), central foveal thickness (CFT) and the incidence of UVL. Inclusion criteria were a detailed clinical documentation and a minimum follow-up (FU) of 2 months after SOR. If available, CFT values were analysed before, during, and after SO.

**Results:**

Overall, 37 eyes received ppV with SO for macula-on RRD. In 25 eyes (68%), SOR was performed within 60 days. Due to organizational issues (Covid, capacity) or patient-related factors (cancellation, illness), SO remained for longer than 60 days in 12 eyes (32%). Primary reattachment was achieved in all eyes. In the short-term group SOR was after 36 days (SD 13.4) and in the long-term cohort after 92 days (SD 20.9). UVL (≥ 2 Snellen lines BCVA loss without correlate) was significantly more common in the long-term (5/12) than in the short-term SO group (0/25) (*p* = 0,002). There were no differences in intra -and postoperative parameters (cut-suture time, IOP, CFT). Eyes with long-term SO had a final BCVA of logMAR 0.5 (SD 0.34) compared to a preoperative BCVA of logMAR 0.3 (SD 0.21). In eyes with short-term SO the BCVA before surgery (0.3; SD 0.29) and after SOR (0.3; SD 0.28) were comparable. The final BCVA after SOR was significantly better after short-term than after long-term tamponade (*p* = 0.033).

**Conclusion:**

Eyes with a shorter SO tamponade (≤ 60 days) had better postoperative BCVA, while 42% in the long-term group (> 60 days) experienced UVL Taking into account the limitations of our small cohort, the risk of UVL during SO tamponade should be considered, especially in macula-on cases. Currently, an early SOR is the only modifiable surgical approach to address this adverse event.

**Trial registration:**

The study was approved by the local ethics committee on 31st of October 2025 (Ethikkommission der Universität Regensburg, Votum 254385104) and was conducted in accordance with the ethical standards of the Declaration of Helsinki.

## Introduction

Unexplained visual loss (UVL) associated with a silicone oil (SO) endotamponade is a serious complication following rhegmatogenous retinal detachment (RRD) surgery. While most eyes with an RRD are treated with pars plana vitrectomy (ppV) and gas, only few cases undergo a primary SO tamponade. Therefore, the condition is rare and has only been described in case series so far [1]. Especially in eyes undergoing ppV for a macula-on RRD with good preoperative vision the risk of irreversible, profound SO-associated visual loss is present with a reported incidence of up to 30–50% [2–4]. The pathophysiology of UVL remains unclear. Mechanisms that are being discussed include the role of SO emulsification, the possible toxicity of SO with subsequent macular dysfunction or metabolic effects due to altered electrolyte levels in the vitreous cavity [1, 5]. Lately the impact of mechanical damage to the foveal region due to a compression effect of the SO bubble in prone position has been mentioned [6]. UVL is usually defined as loss of at least 2 Snellen lines without any clinical explanation, which occurs during or after a SO tamponade [7]. Although, by definition, no obvious structural changes are visible, several alterations during or after SO filling have been described using multimodal imaging like a reduced central foveal thickness (CFT), the thinning of inner retinal layers, an altered central microstructure with flattening of the foveal contour or microvascular changes with reduced vessel density [6, 8–11]. Recently, there have been indications of a correlation of UVL and the presence of microstructural changes with the timing of the SO removal (SOR) [11–13]. Up to now, the duration of the SO tamponade is the only factor that can be meaningfully influenced by surgical intervention. In light of increasing cases of UVL, we established a new protocol to shorten the duration of SO tamponade in cases with macula-on RRD and good functional prognosis. This study investigates the effect of shortened tamponade (≤ 60 days) compared to a long-term SO tamponade (> 60 days) in terms of anatomical and functional outcome in a real-life clinical setting.

## Methods

All eyes with a macula-on RRD, which had ppV with a primary SO tamponade between 2020 and 2025 were retrospectively assessed. The VR procedures were performed by 5 surgeons with experience in at least 1.000 ppVs. Due to the individual nature of RRD, it was impossible to standardize the surgery; however, in every case the same VR machine /pack (Alcon Constellation^®^) and the same SO viscosity (5.000 centistokes) were used. Eyes with a short-term SO tamponade (≤ 60 days) were compared to a longer duration between initial surgery and SOR (> 60 days). The aim was to compare anatomical and functional outcome depending on the SO tamponade duration.

Therefore, the medical charts were reviewed regarding epidemiologic characteristics (sex, age, ocular history) and clinical features e.g. time from diagnosis to surgery, macular status, best corrected visual acuity (BCVA), intraocular pressure (IOP) and central foveal thickness (CFT) measured by a 6-line macular star by optical coherence tomography imaging (OCT; Spectralis©, Heidelberg Engineering), before, during and after SO tamponade. The surgical protocols were used to asses cut-suture-time, the type of SO, the use of laser-/cryopexy and any intraoperative adverse events. Cases with rigorous clinical documentation after primary SO tamponade for Macula-on RRD with pre- and postoperative BCVA, surgical protocols, fundus sketches, IOP values, and a minimum follow-up (FU) of 2 months after SOR met our inclusion criteria. Eyes with a history of trauma or glaucoma, proliferative vitreoretinopathy (PVR) grade C or higher or any previous intraocular surgery apart from uncomplicated cataract surgery were excluded.

The BCVA values before, during (allowing for hyperopic shift due to SO) and after the SO tamponade were converted to logMAR for statistical calculation. The event of UVL was defined by a loss of at least 2 Snellen lines with unremarkable postoperative central OCT scan analysed by two graders unmasked to tamponade duration.

The analysis was completed with SPSS statistics 25 (IBM, USA). Categorical variables were described using frequency counts and percentages, while continuous variables were assessed using means and standard deviations (SD). The exact Fisher’s test was used to determine the association between two categorical variables. Continuous variables were compared using the Mann-Whitney-U-Test or the Wilcoxon Test. For all statistical tests a p-value < 0.05 was considered significant.

## Results

Overall, 37 eyes with a primary SO tamponade due to a macula-on RRD were identified to have vitreoretinal (VR) surgery between 01/2020 and 12/2025. The indication for the use of SO were the presence of a giant retinal tear (*n* = 22), schisis-related RRD (*n* = 7), inferior breaks (*n* = 3), long-standing RRD (*n* = 2), or hereditary vitreoretinopathies like Stickler’s (*n* = 2). In 25 eyes (68%) SOR took place within 60 days after the primary surgery. In 12 cases (32%) the SO tamponade remained longer than 60 days in situ due to either organisational issues (Covid situation, limited capacity) or patient-related circumstance (illness, rescheduling, personal preference). The perioperative parameters of the short and long-term SO tamponade groups are compared below regarding functional and anatomical outcome.

### Baseline characteristics and surgical approach

37 eyes (57% right) of 36 patients (75% male) were analysed. The mean age at initial presentation was 58 years (SD 14.6; range 19–85). On average, patients had noticed visual symptoms for 14 days (SD 21.0; range 1–90). The interval between the diagnosis of RRD and VR surgery was 1.4 days (SD 2.29; range 0–13). The RRD affected on average 5 clock hours (SD 1.9; range 2–11) with inferior breaks below 4 and 8 o’clock in 12 eyes (32%). The baseline characteristics did not differ between the short and long-term group. In Table 1 the baseline data are itemised in detail (see Table 1).

**Table 1:** Baseline characteristics of 37 cases with primary silicone oil (SO) for macula-on rhegmatogenous retinal detachment (RRD)

	Short term SO (*n* = 25, 68%)	Long term SO (*n* = 12, 32%)	*p*-value
site (right)	13 (52%)	8 (67%)	0.491
sex (male)	20 (80%)	8 (67%)	0.432
age (years)	57 (SD 16.3; 95% CI 50, 64)	62 (SD 10.5; 95% CI 55, 68)	0.558
duration of symptoms (days)	11 (SD 16.9; 95% CI: 3, 19)	19 (SD 27.8) 95% CI: 2, 41)	0.200
diagnosis- surgery (days)	1.2 (SD 1.46; 95% CI: 0.6, 1.8)	2.0 (SD 3.46; 95% CI: 0.2, 4.2)	0.235
RRD clock hours	6 (SD 2.0; 95% CI 5, 6)	5 (SD 1.3; 95% CI 4,5)	0.110
inferior breaks	17 (68%)	6 (50%)	0.451
lens status (phakic)	9 (36%)	6 (50%)	0.488

### Intraoperative parameters

All 37 eyes underwent a primary standard 23 Gauge (G) or 25 Gauge ppV (from 2025 onwards) with reattachment of the retina using perfluorocarbon liquid (PFCL: F-Decalin©, Fluoron), endolaser and/or cryopexy of the retinal breaks and 5000 centistokes SO tamponade (Oxane© Bausch &Lomb, Siluron©, Fluoron or RT SlL-OL 5000, Zeiss©) under general anaesthesia. 15 eyes were phakic with a mean spherical equivalent of -1.3 D (SD 2.53; range − 4.25 - +1.25). 22 eyes already had a history of cataract surgery. In nearly all phakic eyes (14/15) a phakovitrectomy was performed. A 19-year-old male with RRD was left phakic and has not developed any lens opacity to date. The SOR was performed via a second ppV using hybrid 25/23 trocars and a temporary air (34/37) or gas (3/37) tamponade. In the short-term group the SO was removed after an average of 36 days (SD 13.4; range 4–60). In the long-term group the SOR was performed after a mean of 92 days (SD 20.9; range 61–131). In Table 2 the intraoperative data is listed in detail (see Table 2). No differences were found with regard to intraoperative data (cut-suture time, cryo-/laserpexy) comparing the both groups. Additionally, no specific intraoperative complications were recorded in the protocols.

**Table 2:** Intraoperative parameters of 37 eyes with ppV and primary silicone oil (SO) tamponade

	Short term SO (*n* = 25, 68%)	Long term SO (*n* = 12, 32%)	*p*-value
cut-suture-time [min]	55 (SD 15.4)	53 (SD 18.7)	0.364
laser spots	801 (SD 254.1)	719 (SD 330.2)	0.295
360° laser	16 (64%)	9 (75%)	0.491
cryopexy	13 (52%)	8 (67%)	0.716
phacovitrectomy	8 (32%)	6 (50%)	0.470
trocar gauge (23/25)	14/11	10/2	

### Perioperative intraocular pressure

The minimum and maximum IOP values of all eyes during SO tamponade were 11 mmHg (SD 3.3; range 4–17) and 21 mmHg (SD 5.7; 14–34) respectively. There was no significant difference in IOP between the eyes with short compared to long-term tamponade duration. There was also no association between IOP levels and the incidence of UVL. 4 of 25 eyes in the short-term and 1 of 12 eyes in the long-term group developed elevated IOP (> 21 mmHg) in the period between first ppV and SOR, which was controlled by topical antiglaucomatous therapy. Table 3 lists the comparison of the perioperative IOD depending on the SO duration (see Table 3).

**Table 3:** Perioperative intraocular pressure (IOP) during silicone oil (SO) tamponade

	Short term SO (*n* = 25, 68%)	Long term SO (*n* = 12, 32%)	*p*-value
Minimum IOP during SO [mmHg]	11 (SD 3.3)	11 (SD 3.5)	0.929
Maximum IOP during SO [mmHg]	23 (SD 9.6)	21 (SD 4.9)	0.825

### Perioperative central foveal thickness

**Fig. 1:**
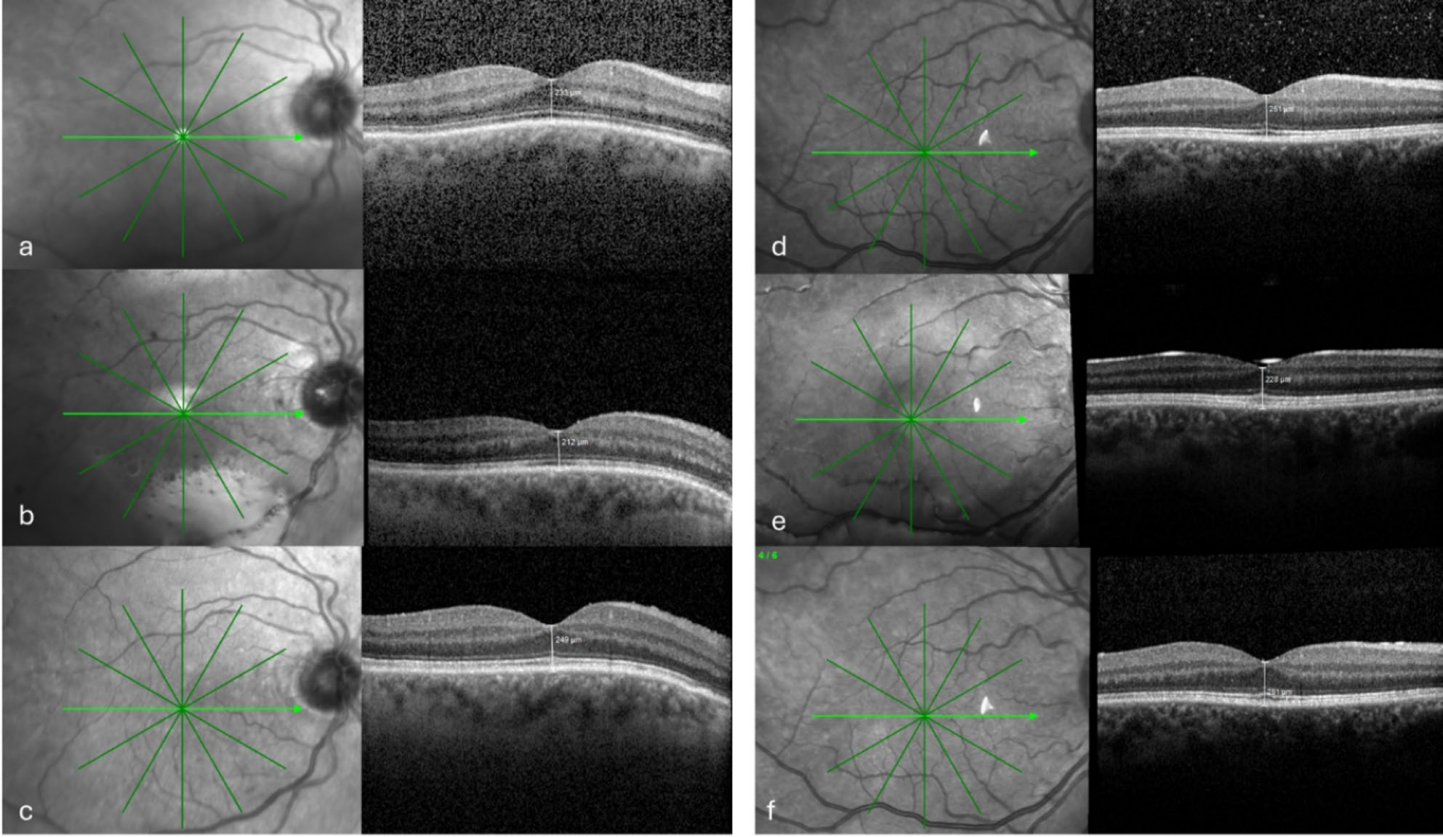
Optical coherence tomography scans of 2 eyes with macula-on rhegmatogenous retinal detachment before silicone oil (SO) tamponade (a/d), during SO with flattening of the foveal contour (b/3) and after SO removal (c/f) with regression to baseline morphology

Due to the urgency of macula-on RRD repair, a preoperative OCT measurement was not available in all cases. The mean CFT of all eyes with available OCT data (19/37) was 251 μm (SD 51.1; range 198–432) before surgery, 236 μm (SD 41.0; range 193–363) during SO tamponade and 253 (SD 40.9; range 188–368) at the final FU after SOR. Eyes with a macular edema (*n* = 5) or epiretinal membrane (*n* = 2) at the final visit were excluded from the CFT analysis. The decrease of CFT from baseline to the interval during SO tamponade (*p* = 0.015) as well the increase of the CFT after SOR were statistically significant *p* = 0.020). The mean CFT before, during and after the SO did not differ between the short and the long-term group (see Table 4). In 9 of 19 eyes (47%) with OCT imaging also a qualitative flattening of the foveal contour was seen during the SO tamponade, which recovered to the preoperative morphology after SOR (see Fig. 1). This reversible flattening of the foveal microstructure did not correlate with worse BCVA values.

**Table 4:** Perioperative central foveal thickness (CFT) before, during and after silicone oil (SO)

	Short term SO (*n* = 12)	Long term SO (*n* = 7)	*p*-value
CFT before SO [µm]	237 (SD 32.1)	274 (SD 70.4)	0.234
CFT during SO [µm]	228 (SD 28.7)	251 (SD 56.0)	0.606
CFT after SO [µm]	251 (SD 45.2)	257 (SD 35.2)	0.832

### Functional outcome

6 eyes with a preoperative vitreous hemorrhage (VH) were excluded from the baseline BCVA analysis. On average, the preoperative BCVA of all eyes without VH before the SO tamponade was 0.3 logMAR (SD 0.26; range 0–1.0). One 85-year-old female with a schisis detachment presented with a preoperative logMAR BCVA of 1.0 due to an advanced cataract. During the SO filling the BCVA declined to a mean of 0.4 logMAR (SD 0.28; range 0.1–1.0) and recovered again to 0.3 logMAR (SD 0.32; range 0–1.0) after SOR. The change from baseline BCVA to a reduced BVCA during SO (*p* = 0.046) as well as the recovery after SOR compared to the BCVA during SO were statistically significant (*p* = 0.016). In Table 5 the BCVA values of both groups are recorded in detail (see Table 5). In the short-term group with a mean SO duration of 36 days (SD 13.4) the BCVA was stable with 0.3 logMAR before SO (SD 0.29) and 0.3 logMAR (SD 0.28) after SOR, while in the long-term group with a mean SO duration of 92 days (SD 20.9) the visual acuity declined from 0.3 logMAR (SD 0.21) at baseline to 0.5 logMAR (SD 0.34) after SOR. The BCVA values of the short and long-term SO group are depicted in Fig. 2 (see Fig. 2). The final BCVA after the SO tamponade was statistically significantly better in the group with a quick SOR (within 60 days) compared to the group with a deferred SOR (after 60 days) (*p* = 0.033). In eyes with an earlier SOR no case of UVL was seen. The event of UVL (BCVA loss of ≥ 2 Snellen lines) was significantly more frequent in the long-term group (5/12) than in the short-term group (0/25) (*p* = 0,002, Chi-Quadrat-Test). Additionally, the SO tamponade duration was significantly shorter in the group without UVL compared to eyes with visual loss (*p* = 0.002; Mann-Whitney-U Test). All events of UVL were documented during the SO tamponade. NO UVL happened after the SOR procedure.

**Table 5:** Pre- and postoperative best corrected visual acuity (BCVA) and incidence of unexplained visual loss compared to SO tamponade duration

	Short term SO (*n* = 25, 68%)	Long term SO (*n* = 12, 32%)	*p*-value
SO tamponade duration (days)	36 (SD 13.4; 95% CI 32, 42)	92 (SD 20.9; 95% CI 79, 105)	< 0.001
logMAR BCVA before SO	0.5 (0.63; 95% CI 0.3, 0.8)	0.5 (SD 0.62; 95% CI 0.6, 0.8)	0.891
logMAR BCVA before SO #	0.3 (SD 0.29; 95% CI 0.2, 0.5)	0.3 (SD 0.21; 95% CI 0.1, 0.4)	1.000
logMAR BCVA during SO	0.4 (SD 0.26; 95% CI 0.2, 0.5)	0.5 (SD 0.28; 95% CI 0.4, 0.7)	0.036
logMAR BCVA after SO	0.3 (SD 0.29; 95% CI 0.1, 0.4)	0.5 (SD 0.34; 95% CI 0.3, 0.7)	0.033
Unexplained visual loss	0 (0%)	5 (42%)	0.002

# eyes with vitreous haemorrhage at initial presentation excluded

**Fig. 2:**
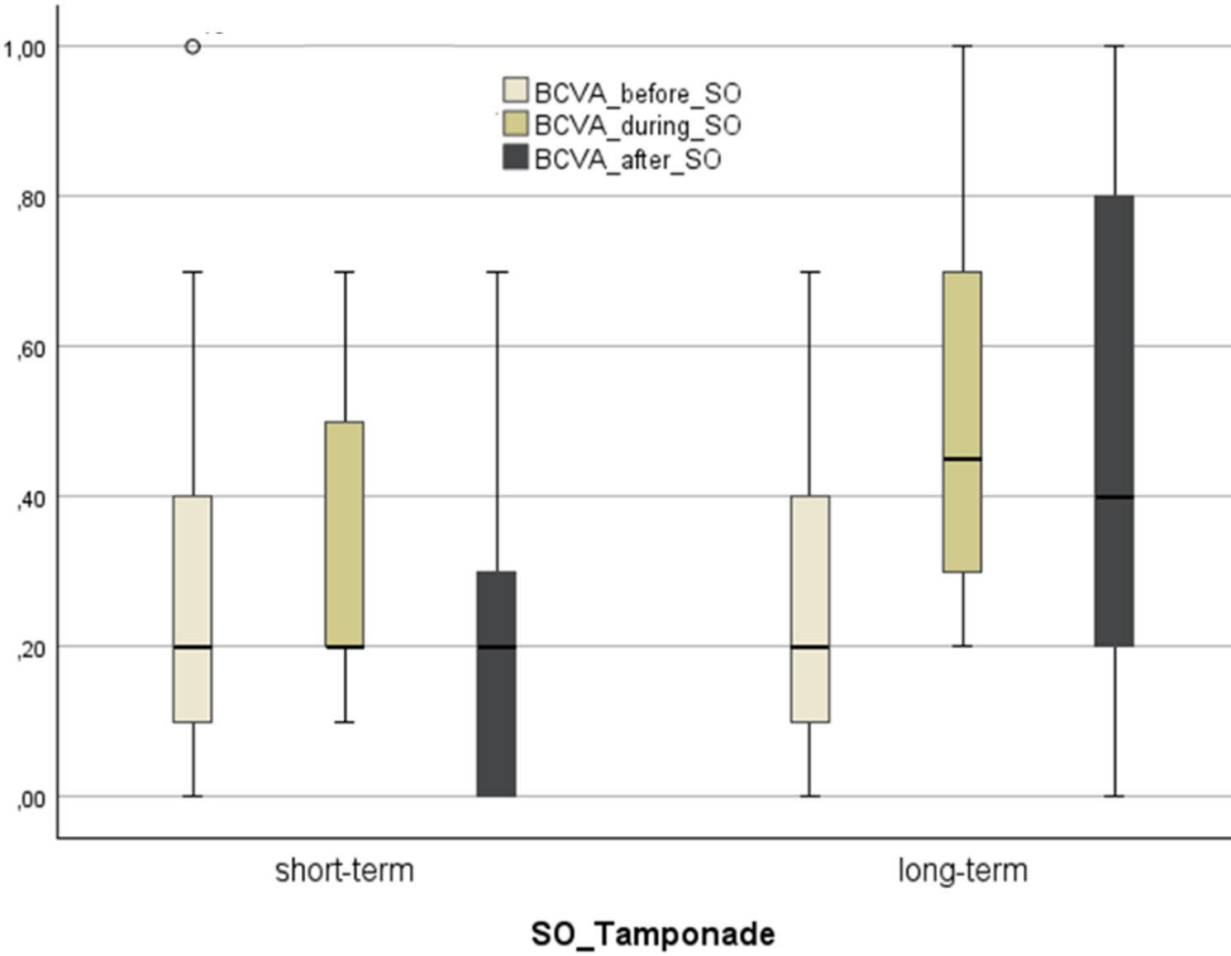
Best-corrected visual acuity (BCVA) of the short-term and the long-term silicone oil (SO) tamponade group before, during and after SO (# eyes with vitreous haemorrhage at initial presentation excluded)

### Anatomical outcome and postoperative complications

Primary retinal attachment was achieved in all eyes. After SOR no redetachment occurred within the mean FU period of 7 months (SD 6.7; range 2–24). 13 of 37 eyes (35%) presented with postoperative complications like secondary glaucoma (*n* = 5; 14%), macular edema (ME) (*n* = 5; 14%), secondary epiretinal membranes (*n* = 2; 5%) or corneal decompensation (*n* = 1). No difference regarding the mentioned postoperative complications was noticed between the short and the long-term SO group.

## Discussion

Recently, the risk of SO-related visual loss after RRD repair has got into the focus of VR surgeons. In view of increasing descriptions of this phenomenon with irreversible, functional loss during or after a SO tamponade, we established a new clinical protocol of an earlier SOR within 60 days in cases with macula-on RRDS in 2020. Since the new schedule could not be implemented in all cases (for the reasons mentioned above), we were able to retrospectively compare cases with an early and a deferred SOR. We are aware that the 60-day limit is an artificial cut-off. In the literature, the minimum tamponade duration during which UVL was observed is 76 days [3]. Therefore, a “safe timeline” of 60 days seemed reasonable to us.

The baseline characteristics and intraoperative parameter did not differ between these two groups. Also, the perioperative IOP values showed no clinically apparent abnormalities. However, it should be noted that in the literature an association of uncontrolled IOP levels and the occurrence of UVL has been stated [7, 14, 15], which was not confirmed in our cohort. Li et al. also found a correlation of increased IOP and prolonged SO tamponade [13]. Generally, in clinical routine regular IOP monitoring should be recommended for all eyes with a SO tamponade.

Regarding the anatomical outcome (no redetachment within mean FU of 7 months) our two groups did not differ, while in the long-term group a high rate of SO-related visual loss was recorded (42%). which also resulted in poorer BCVA at the final visit. Simultaneously, the functional outcome was significantly better in the group with an earlier SOR. The association between SO tamponade duration and worse functional outcome has been noted in several reports [2, 15, 16]. Roca et al. found a higher incidence of UVL in eyes with a longer (14.8 months) compared to a shorter tamponade (9.3 months) [15]. This study had a high percentage (66%) of macula-off RRDs and an overall very long tamponade duration. Here, the incidence of UVL was 7%, which is comparable to other series ranging from 3 to 11% in macula-off conditions [3, 7, 12]. In macula-on cases higher incidences of vision loss have been described [2–4, 17]. Tode et al. found a persistent BCVA loss in 3/15 (20%) eyes with macula-on RRD during SO tamponade within the first three months. Our data with an incidence of 42% of UVL is consistent with reports of a 20–50% incidence of SO-related visual loss, when including macula-on situations only [2, 3, 17]. However, it should be noted that our results are based on a small and non-randomized cohort.

Regarding the effect of the tamponade duration, Park et al. assessed in a large cohort of 1017 eyes with RRD significantly better BCVA and better vision recovery after SOR in eyes with shorter SO duration compared to a longer tamponade [18]. This study lacks the report of the macula status and the overall tamponade duration was quite long with an average of 6.68 ± 11.39 months [18]. Alongside, Chen et al. reported significantly better postoperative BCVA in 67 eyes with macula-off RRD when comparing short-term (2 months) to a long-term SO tamponade with also less UVL in the short-term group [12]. Furthermore, a recent series of 66 RRDs by Moussa et al. found a better microperimetric retinal sensitivity, but a similar postoperative BCVA when comparing early (2 month) with delayed (6 months) SOR. Here, only macula-off cases were assessed [18]. Recently, Desideri et al. reported an association of the duration of SO tamponade and the presence of ME in a cohort of 114 eyes with a 56% rate of ME and an overall quite long tamponade duration of 665 day (SD 580) in the ME group [19]. In a cohort of 65 cases, Er et al. also noticed a significantly higher incidence of 50% ME after RRD repair with a SO duration of ≥ 6 months compared to eyes with shorter tamponade and therefore recommended SOR within 3 months after primary surgery [20]. Other risk factors influencing the occurrence of postoperative ME are macula off status, the presence of preoperative PVR and RRD recurrence [21]. In our data, only 14% of the eyes developed ME, which could be related to the shorter tamponade duration and the other mentioned factors, which were absent in our cohort.

Several authors also described qualitative and quantitative changes of central retinal microstructure during SO tamponade, which occasionally correlated with the tamponade duration. Christensen et al. were the first to report a profound thinning of the inner retinal layers (but no significant CFT reduction) in a case series of 16 macula-on RRDs in eyes after SOR compared to a gas tamponade with also correlated with poorer visual outcome [8]. The duration of SO tamponade was quite long in this series with nearly 5 months (range 94–308 days) without any association between the prolonged tamponade and final BCVA, whereas the postoperative BCVA was in general reduced to 0.1 in 30% of the eyes after SOR [8]. Tode et al. noted thinning of the inner retinal layers on 6/9 eyes compared to healthy fellow eyes, but no significant reduction in CFT after SOR [17]. No exact dates of SO duration were stated in this series [17]. Li et al. found a correlation of thinning of CFT and inner retinal layers with the tamponade duration [13]. However, this study lacks preoperative CFT values and included mainly macula-off cases. In another case series of 44 macula-on detachments by Scheerlinck et al. eyes with UVL and a mean SO tamponade of 18 weeks had significantly reduced inner retinal layers compared to eyes without UVL and shorter SO tamponade (13 weeks) [2]. In our series of 22 macula-on RRDs the CFT was reduced in all eyes during SO filling (with recovering after SOR and the incidence of UVL was 50% with a tendency to a longer SO tamponade in UVL cases (20 weeks) compared to non-UVL cases (17 weeks), which was statistically not significant [4]. Al-Sheri et al. reported a series of 26 macula-on RRDs with SO filling, which compared to 32 eyes after gas showed generalised central retinal thinning (not only affecting the inner layers) along with a flattened foveal configuration in 20% of the eyes, which resolved after SOR. In this cohort, no effect of the duration of the SO tamponade (mean of 8 months) on the altered foveal structure or postoperative BCVA was found [10]. In a metanalysis of 11 articles by Ghanbari et al. also a reduced CFT during SO with recovery after SOR to fellow eye values was found. Here, no data of preoperative CFT is available and cases were only compared to eyes treated with gas or healthy fellow eyes [22]. Recently, Ewais et al. found in their cohort of 64 macula-off cases, a distinct central and parafoveal thinning during the SO filling [11]. This is in accordance with the hypothesis of a flattened curvature of the central macula by mechanical compression of the SO bubble [6]. Ewais et al. also described that the recovery of the decreased CFT and parafoveal thickness (PFT) after SOR correlated with the duration of the tamponade. While the CFT and PFT of eyes with a shorter SO tamponade (3–4 months) recovered back to baseline values, the cases with a longer (6–8 months) SO filling showed a slight increase after SOR, but were still significantly reduced compared to before SO [11]. The BCVA after SOR was also significantly better in the shorter tamponade group compared to the long-term group. However, it should be noted that this study only included macula-off conditions and that the baseline CFT values were taken from the fellow eye.

In our cohort, we were able to exclusively analyse macula-on conditions with access to preoperative OCT scans. Here, our results are limited because a preoperative scan was only available for half of the eyes (19/37). We registered a flattened curvature of the fovea in 9/19 (47%) of the available OCT scans during SO, which was reversible in all cases after SOR and did not correlate with a worse functional outcome. The question of whether the flattening visible on the OCT scans is actually anatomically present or merely the result of an altered optical measurement due to the SO tamponade should be taken into account. We know from literature that OCT-based biometry overestimates the axial length in SO-filled eyes along with a longer vitreous cavity length possibly due to a higher refractive index of SO with a slightly reduced speed of light passing through the endotamponade, which would explain a “stretching” of the OCT image [23, 24]. Overall, we found a significantly recued CFT during SO filling, which returned to preoperative values after SOR. In accordance with our results, several authors consistently described reduced CFT during the SO tamponade, which recovered after SOR [4, 10]. In some reports the rate of recovery showed also a negative correlation with the tamponade duration [10]. Regarding functional outcome, the worse BCVA after a longer SO tamponade compared to a shorter SO filling has constantly been reported [2, 11, 12]. Here, the effect of the duration of SO tamponade on postoperative BCVA appears to be more evident when a short tamponade is compared with longer intervals, whereas with tamponades that are already quite long (> 4 months), no difference in the occurrence of SO-associated visual loss becomes clinically apparent [7, 8].

The key limitations of our study are that our assumptions rely on small, non-randomised groups with few UVL events (0/25 vs. 5/12), limited follow-up and with preoperative OCT scans only available in half of the eyes. Furthermore, the two groups were not numerically balanced, and non-random factors that led to a prolonged SO tamponade may have also influenced the outcome. In principle, a prospective study would be extremely difficult due to the rarity of a primary SO tamponade in macula-on conditions and the individual endotamponade selection based on intraoperative findings. In view of the high incidence of UVL, general caution is advised when using a SO tamponade in macula-on RRDs. Based on the literature and our own data, it is generally not possible to determine the duration of a SO tamponade that can be considered low-risk in terms of visual loss. Therefore, the duration of the SO tamponade should be kept as short as possible. Here, the literature and our own data give support, that the anatomical results (reattachment rates) are comparable for shorter (≤ 2 months) and longer (> 2 months) SO tamponade durations [12, 19, 25]. In clinical practice, a SOR after 6–8 weeks appears to be a realistic approach when the reattached retina is stable. A longer duration of the SO tamponade should be reasonably justified.

## Data Availability

The data that support the findings of this study are available from teresa.barth@ukr.de. Restrictions apply to the availability of these data. Because of the low sample size, case-based assumptions could be made by individual clinical data, and patients could be identified. Therefore, data are only available from the authors upon reasonable request and with permission of the local ethics committee.

## Abbreviations

BCVA: Best corrected visual acuity
CFT: Central foveal thickness
IOP: Intraocular pressure
ME: Macular edema
OCT: Optical coherence tomography
PFCL: Perfluorocarbon liquid
PFT: Parafoveal thickness
ppV: pars-plana-vitrectomy
PVR: Proliferative vitreoretinopathy
RRD: Rhegmatogenous retinal detachment
SO: Silicone oil
SOR: Silicone oil removal
UVL: Unexplained vision loss
VH: Vitreous haemorrhage
VR: Vitreoretinal

## References

[1] Januschowski K, Rickmann A, Smith J, Pastor-Idoate S, Pastor JC. Vision loss associated with silicone oil endotamponade in vitreoretinal surgery - a review. Graefes Arch Clin Exp Ophthalmol. 2024;262(11):3453–63. 10.1007/s00417-024-06520-y. Epub 2024 Jun 18. 38888804 10.1007/s00417-024-06520-y

[2] Scheerlinck LM, Schellekens PA, Liem AT, Steijns D, Leeuwen Rv incidence, risk factors, and clinical characteristics of unexplained visual loss after intraocular silicone oil for macula-on retinal detachment. Retina. 2016;36(2):342–50. 10.1097/IAE.0000000000000711. 26308530 10.1097/IAE.0000000000000711

[3] Moya R, Chandra A, Banerjee PJ, Tsouris D, Ahmad N, Charteris DG. The incidence of unexplained visual loss following removal of silicone oil. Eye (Lond). 2015;29(11):1477–82. 10.1038/eye.2015.135. Epub 2015 Aug 7. 26248526 10.1038/eye.2015.135 PMC4815673

[4] Barth T, Helbig H, Maerker D, Gamulescu MA, Radeck V. Unexplained visual loss after primary pars-plana-vitrectomy with silicone oil tamponade in fovea-sparing retinal detachment. BMC Ophthalmol. 2023;23(1):75. 10.1186/s12886-023-02823-6. Erratum in: BMC Ophthalmol. 2023;23(1):105. doi: 10.1186/s12886-023-02861-0. 36829157 10.1186/s12886-023-02823-6 PMC9951486

[5] Scheerlinck LME, Kuiper JJW, Liem ATA, Schellekens PAWJF, van Leeuwen R. Electrolyte composition of retro-oil fluid and silicone oil-related visual loss. Acta Ophthalmol. 2016;94(5):449–53. 26806559 10.1111/aos.12959

[6] Lee J, Cho H, Kang M, Hong R, Seong M, Shin Y. Retinal Changes before and after Silicone Oil Removal in Eyes with Rhegmatogenous Retinal Detachment Using Swept-Source Optical Coherence Tomography. J Clin Med. 2021;10(22):5436. 10.3390/jcm10225436. 34830717 10.3390/jcm10225436 PMC8619201

[7] Oliveira-Ferreira C, Azevedo M, Silva M, Roca A, Barbosa-Breda J, Faria PA, Falcão-Reis F, Rocha-Sousa A. Unexplained Visual Loss After Silicone Oil Removal: A 7-Year Retrospective Study. Ophthalmol Ther. 2020;9(3):1–13. 10.1007/s40123-020-00259-5. Epub 2020 Aug 5. 32399859 10.1007/s40123-020-00259-5 PMC7406612

[8] Christensen UC, la Cour M. Visual loss after use of intraocular silicone oil associated with thinning of inner retinal layers. Acta Ophthalmol. 2012;90(8):733–7. 10.1111/j.1755-3768.2011.02248.x. Epub 2011 Sep 13. 21914150 10.1111/j.1755-3768.2011.02248.x

[9] Lee JY, Kim JY, Lee SY, Jeong JH, Lee EK. Foveal Microvascular Structures in Eyes with Silicone Oil Tamponade for Rhegmatogenous Retinal Detachment: A Swept-source Optical Coherence Tomography Angiography Study. Sci Rep. 2020;10(1):2555. 10.1038/s41598-020-59504-3. 32054939 10.1038/s41598-020-59504-3 PMC7018724

[10] Al-Shehri AM, Aljohani S, Aldihan KA, Alrashedi MJ, Alrasheed S, Schatz P. Effect of silicone oil versus gas tamponade on macular layer microstructure after pars plana vitrectomy for macula on rhegmatogenous retinal detachment. BMC Ophthalmol. 2024;24(1):119. 10.1186/s12886-024-03377-x. 38486220 10.1186/s12886-024-03377-x PMC10938769

[11] Ewais WA, Ali LS, Aboalazayem FM. Impact of duration of silicone oil tamponade on foveal and parafoveal thickness in rhegmatogenous retinal detachment: a retrospective cohort study. Int Ophthalmol. 2024;44(1):167. 10.1007/s10792-024-03096-8. 38565753 10.1007/s10792-024-03096-8 PMC10987340

[12] Chen J, Ni Y, Li M, Ge X, Sheng A, Liu H. Effect of shorter silicone oil tamponade time on visual function recovery and associated factors in patients with rhegmatogenous retinal detachment. BMC Ophthalmol. 2026;26(1):106. 10.1186/s12886-026-04625-y. 41593568 10.1186/s12886-026-04625-y PMC12924583

[13] Li J, Wu W, Huang C, Ma Y, Gu P, Lin Q, Liu J, Kea P, Yuan Y, Lin L, Shen P, Li J, Feng S. Postoperative Outcomes of 1-Month Silicone Oil Tamponade in Rhegmatogenous Retinal Detachment: A Multicenter Study. Ophthalmic Res. 2025;68(1):333–41. 10.1159/000546255. Epub 2025 May 19. 40388895 10.1159/000546255

[14] Marti M, Walton R, Böni C, Zweifel SA, Stahel M, Barthelmes D, Increased intraocular pressure is, a risk factor for unexplained visual loss during silicone oil endotamponade. Retina. 2017;37(12):2334–40. 10.1097/IAE.0000000000001492. 28098737 10.1097/IAE.0000000000001492

[15] Roca JA, Wu L, Berrocal M, Rodriguez F, Alezzandrini A, Alvira G, et al. Un-explained visual loss following silicone oil removal: results of the Pan American Collaborative Retina Study (PACORES) Group. Int J Retina Vitreous. 2017;3:26. 28748109 10.1186/s40942-017-0079-6 PMC5523152

[16] Tode J, Purtskhvanidze K, Oppermann T, Hillenkamp J, Treumer F, Roider J. Vision loss under silicone oil tamponade. Graefes Arch Clin Exp Ophthalmol. 2016;254(8):1465–71. 27278374 10.1007/s00417-016-3405-z

[17] Park W, Kim M, Kim RY, Kim JY, Kwak JH, Park YG, Park YH. Long-term visual prognosis and characteristics of recurrent retinal detachment after silicone oil removal. PLoS ONE. 2023;18(2):e0265162. 10.1371/journal.pone.0265162. PMID: 36753472; PMCID: PMC9907833. 36753472 10.1371/journal.pone.0265162 PMC9907833

[18] Moussa AMY, Albalkini AS, Omar HA, Macky TA, Khattab A, Abdullatif AM. Effect of silicone oil removal timing on functional and structural outcomes in primary rhegmatogenous retinal detachment. Int J Retina Vitreous. 2026;12(1):44. 10.1186/s40942-026-00809-2. 41715238 10.1186/s40942-026-00809-2 PMC13019722

[19] Desideri LF, Aissani M, Bernardi E, Zinkernagel M, Anguita R. Macula edema and silicone oil tamponade: clinical features, predictive factors, and treatment. Retina. 2025. 10.1097/IAE.0000000000004450. 10.1097/IAE.0000000000004450 40068017

[20] Er D, Öner H, Kaya M, Dönmez O. Evaluation of the Effects of Silicone Oil on the Macula with Optical Coherence Tomography in Patients with Rhegmatogenous Retinal Detachment. Turk J Ophthalmol. 2021;51(4):218–24. 10.4274/tjo.galenos.2020.48376. 34461708 10.4274/tjo.galenos.2020.48376 PMC8411287

[21] Bernardi E, Shah N, Ferro Desideri L, Potic J, Roth J, Anguita R. Cystoid Macular Edema Following Rhegmatogenous Retinal Detachment Repair Surgery: Incidence, Pathogenesis, Risk Factors and Treatment. Clin Ophthalmol. 2025;19:629–39. 10.2147/OPTH.S489859. 40007877 10.2147/OPTH.S489859 PMC11853832

[22] Ghanbari H, Kianersi F, Jamshidi Madad A, Dehghani A, Rahimi A, Feizi A, et al. The effect of silicone oil tamponade on retinal layers and choroidal thickness in patients with rhegmatogenous retinal detachment: a systematic review and meta-analysis. Int J Retina Vitreous. 2021;7(1):1–14. 34930505 10.1186/s40942-021-00348-y PMC8691011

[23] Kaiser KP, Jandewerth T, Bucur J, Kohnen T, Lwowski C. Axial length adjustment in eyes with silicone oil endotamponade reduces overestimation by a swept-source optical coherence tomography-based biometer. Clin Exp Ophthalmol. 2024;52(8):833–9. 10.1111/ceo.14418. Epub 2024 Jul 21. PMID: 39034156. 39034156 10.1111/ceo.14418

[24] Zhang J, Han X, Zhang M, Liu Z, Lin H, Qiu X, Huang X, Li T, Lv L, Chen X, Jin G, Tan X, Luo L, Liu Y. Comparison of axial length measurements in silicone oil-filled eyes using SS-OCT and partial coherence interferometry. J Cataract Refract Surg. 2022;48(12):1375–80. 10.1097/j.jcrs.0000000000000996. Epub 2022 Jun 20. 35786813 10.1097/j.jcrs.0000000000000996

[25] Kounatidou NE, Mautone L, Druchkiv V, Spitzer MS, Skevas C. Early versus late silicone oil tamponade removal after rhegmatogenous retinal detachment: a retrospective real world comparative study. Int J Retina Vitreous. 2025;11(1):105. 10.1186/s40942-025-00743-9. 41084092 10.1186/s40942-025-00743-9 PMC12516905

